# Factors that interfere with immediate return to activity following volar locking plate fixation for distal radius fractures

**DOI:** 10.3892/mi.2024.189

**Published:** 2024-08-14

**Authors:** Kenjiro Kawamura, Kiyohito Naito, Takamaru Suzuki, Yasuhiro Yamamoto, So Kawakita, Norizumi Imazu, Muneaki Ishijima

**Affiliations:** 1 Department of Medicine for Orthopaedics and Motor Organ, Juntendo University Graduate School of Medicine, Tokyo 113-8421, Japan; 2 Department of Orthopaedics, Faculty of Medicine, Juntendo University, Tokyo 113-8421, Japan

**Keywords:** distal radial fractures, patient-reported outcome measures, immediate return to activity, post-operative pain, grip strength ratio

## Abstract

In the present study, the clinical findings that interfere with the immediate return to activity following volar locking plate (VLP) fixation for distal radius fractures were investigated. A total of 95 patients who underwent VLP fixation for distal radius fracture between July, 2014 and January, 2022 were divided into a good group (good score and outcome; n=86; 22 males and 64 females; median age, 61 years) and a poor group (poor score and outcome; n=8; 8 females; median age, 63.6 years) according to the quartiles of the disabilities of the arm, shoulder and hand (Q-DASH) score, at 1 month following VLP fixation. The duration from injury to surgery, the direction of fracture dislocation and radiographic parameters [radial inclination (RI), volar tilt (VT) and ulnar variance (UV)] at the time of injury were examined. Radiographic parameters (RI, VT and UV), the range of motion of the wrist joint, grip strength ratio and visual analog scale (VAS) at 1 month following VLP fixation were also examined. These parameters were compared among both groups. Moreover, logistic regression analysis was performed to determine whether these factors were independently associated with a poor Q-DASH score at 1 month following VLP fixation. At the time of injury, fracture displacement was significantly higher in the poor group (VT, -23.8˚; UV, 4.2 mm) than the good group (VT, -6.5˚; P=0.02; UV, 1.3 mm; P=0.01). No differences in the other parameters were observed between the groups. At 1 month following VLP fixation, the grip strength ratio (17.2%) in the poor group was significantly lower than that in the good group (43.8%, P<0.001), while the VAS score (5.6) in the poor group was significantly higher than that in the good group (2.4, P<0.001). Logistic regression analysis revealed that VT and UV at injury (P<0.05), grip strength ratio (P<0.001) and pain (VAS score) (P<0.001) were all independently associated with a poor Q-DASH score. On the whole, the present study suggests that large amounts of fracture displacement, weakness of grip strength and post-operative pain can be factors interfering with the return to activity immediately following VLP fixation.

## Introduction

Distal radius fractures are among the most common fractures encountered in clinical practice (1,2). Conservative treatment is the main treatment, especially in elderly patients (about 70-85% of patients) (3,4). On the other hand, for the purpose of immediate return to activity and daily activities, young patients often opt for volar locking plate (VLP) fixation (5,6). VLP fixation improves stability in the short term (7). VLP fixation has recently gained widespread application in the elderly population (5).

The functional evaluation of the wrist joint is widely used to assess the effectiveness of distal radius fracture treatment. This evaluation typically encompasses objective outcomes and patient-reported outcomes, which are interconnected (8). Patient-reported outcomes are used to evaluate patient pain and directly reflect quality of life (8). The quick disabilities of the arm, shoulder and hand (Q-DASH) score, a widely used patient-reported outcome score of the upper extremity, has been reported to be an indicator of return to activity (9).

An advantage of VLP fixation is the immediate return to activity. However, some patients are unable to return to activity immediately following surgery due to complications associated with VLP fixation, such as infection, tendon injury and screw puncture. Moreover, there have been reports of cases of poor patient-reported outcomes due to pain, swelling and contractures despite the absence of complications associated with VLP fixation (10-12). It is thus critical to identify the factors associated with poor early post-operative patient-reported outcomes in VLP fixation in order to achieve immediate return to activity.

The present study compared post-operative outcomes using the Q-DASH score in the very early post-operative period (1 month following VLP fixation). The present study aimed to identify clinical findings associated with poor outcomes.

## Patients and methods

### Patients

The present study was approved by the Ethics Committee for Medical Research of Juntendo University (Tokyo, Japan; No. E22-0447; date of approval: September 21, 2023). Informed consent was obtained both for participation and publication in the form of opt-out on the web-site, and those who rejected were excluded. The present study was a retrospective study. The patients were subjected to standard clinical practice, including the methods of anesthesia and surgery.

**Figure 1:**
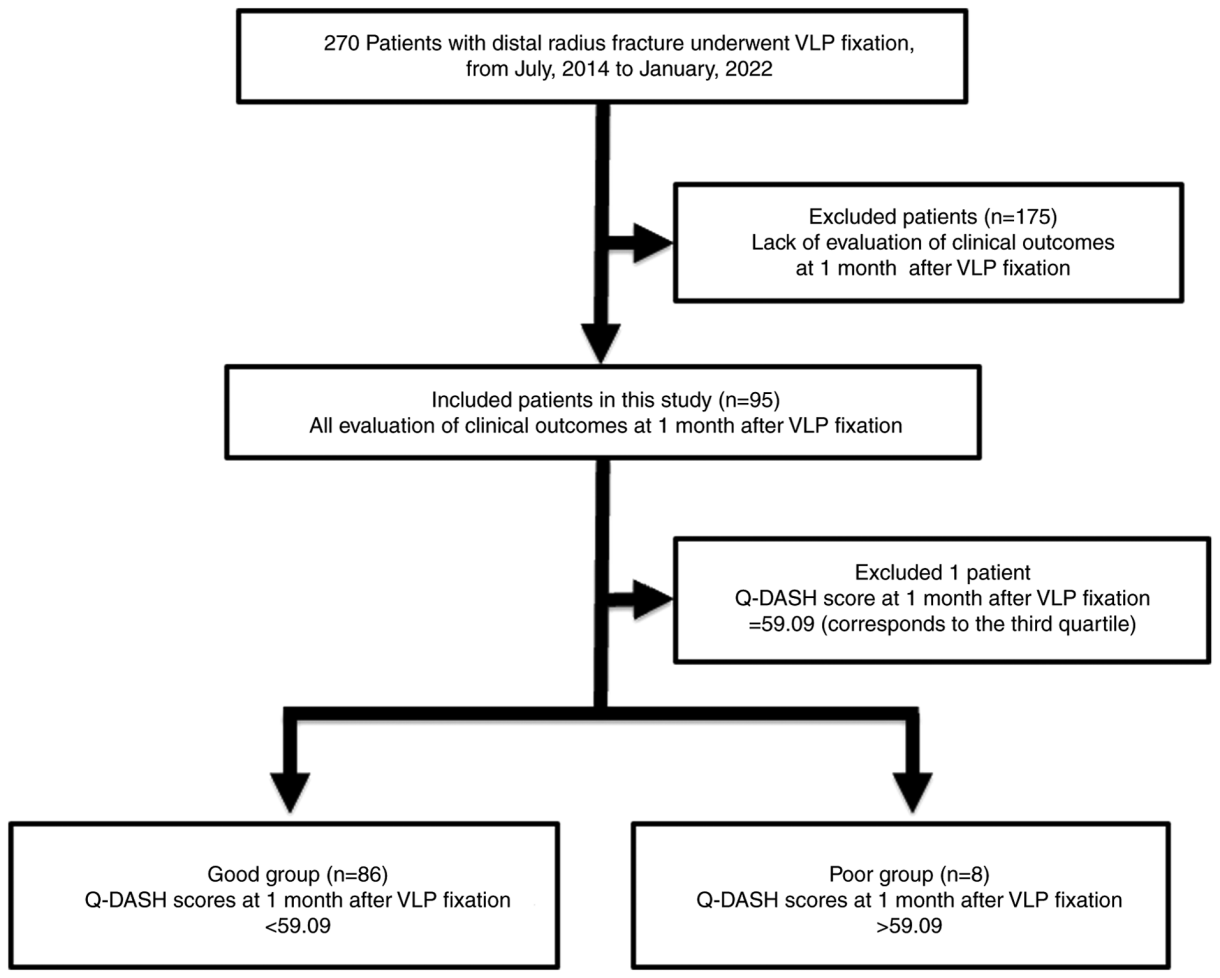
Diagram of the patient selection process. VLP, volar locking plate; Q-DASH score, quick disabilities of the arm, shoulder and hand score.

From July, 2014 to January, 2022, 270 patients with distal radius fracture underwent VLP fixation at Juntendo University Hospital. In the present study, 95 patients (23 males and 72 females; mean age, 61.9±14.7 years; range, 22-87 years) with all evaluation items recorded at 1 month post-operatively were included (Fig. 1). The fracture type was defined according to the Arbeitsgemeinschaft für Osteosynthesefragen (AO) classification: Type A (n=18 patients), type B (n=5 patients) and type C (n=72 patients). There were no complications associated with VLP fixation at 1 month following surgery.

### Division into groups

The Q-DASH score at 1 month following VLP fixation was used as an index of return to activity. A total of 95 patients were divided into groups based on the Q-DASH score for the analysis of factors associated with immediate return to activity. At 1 month after surgery, the mean Q-DASH score was 32.7±18.7 (range, 4.55-84.09). The patients were divided according to the Q-DASH score into good (good score and outcome) and poor (poor score and outcome) groups according to quartiles (median, 40.91; first quartile, 22.73; third quartile, 59.09). The good group consisted of 86 patients with Q-DASH scores <59.09 (mean, 28.6±14.3; range, 4.55-54.55). The poor group consisted of 8 patients with Q-DASH scores >59.09 (mean, 73.0±6.2; range, 65.91-84.09). Of note, 1 patient with a Q-DASH of score of 59.09 (44-year-old male, AO classification type C) was omitted from the analysis (Fig. 1). As regards the treatment of patients within the third quartile, cases in the third quartile may be omitted from the analysis or included in the good or poor group. Fritzsching *et al* (13) used quartiles and omitted the third quartile in their analysis of the prognosis of osteosarcoma when dividing the patients into good or poor groups. According to that study, the statistical analysis was also performed by omitting cases with a Q-DASH score of 59.09, which corresponds to the third quartile, in the present study.

The background of the patients [the evaluation items were as follows: Age, sex, height, weight, body mass index (BMI), injury energy (high energy injury pertains to high energy trauma, such as that sustained by a vehicle accident and falling from a height; low energy injury pertains to low energy trauma, such as that sustained by falling down), AO classification, and Q-DASH score] were investigated and compared between the two groups (Table I).

### Evaluation

The duration from injury to surgery, the direction of fracture dislocation and radiographic parameters [radial inclination (RI), volar tilt (VT) and ulnar variance (UV)] at the time of injury were examined. In addition, at 1 month following VLP fixation, the radiographic parameters (RI, VT and UV), Q-DASH score, wrist joint range of motion (flexion, extension, pronation and supination), grip strength ratio and visual analog scale (VAS) were examined.

### Statistical analysis

Data were analyzed for significant differences through two-group comparisons. Fisher's exact test was utilized for assessing sex, injury energy, the direction of fracture dislocation and AO classification, and the Mann-Whitney U test was used for other endpoints. Factors influencing the poor group were identified. Multivariate analysis (logistic regression analysis) was performed to determine whether the factors were independently associated with poor Q-DASH scores at 1 month post-operatively. Given that the sample size of the study was 95 patients, with only 8 patients in the poor outcome group, two explanatory variables were included in the logistic regression analysis. Data are presented as the mean ± standard deviation. A value of P<0.05 was considered to indicate a statistically significant difference.

## Results

The age of the patients was 61.9±14.7 years in the good group and 63.6±15.2 years in the poor group, with no significant difference between the two groups (Mann-Whitney U test, P=0.95). As regards sex, there were 22 males and 64 females in the good group, and 8 females in the poor group, with no significant difference between the two groups (Fisher's exact test, P=0.19). Height was 158.2±14.7 cm in the good group and 153.0±8.0 cm in the poor group, with no significant difference between the two groups (Mann-Whitney U test, P=0.33). Body weight was 58.6±10.1 kg in the good group and 50.5±8.2 kg in the poor group, indicating that the patients in the poor group had a significantly lower weight than those in the good group (Mann-Whitney U test, P=0.03). BMI was 22.9±2.9 g/m^2^ in the good group and 21.6±3.3 kg/m^2^ in the poor group, with no significant difference between the two groups (Mann-Whitney U test, P=0.22). Injury energy was high (vehicle accident and falling from a height) in 19 cases and low (falling down) in 67 cases in the good group, and high in 2 cases and low in 6 cases in the poor group, with no significant difference between the two groups (Fisher's exact test, not significant). As regards the AO classification, in the good group, 18 patients were classified as type A, 5 patients were classified as type B, and 63 patients were classified as type C; in the poor group, the 8 patients were classified as type C; there was a significantly greater number of type C fractures in the poor group than in the good group (Fisher's exact test, P<0.01). The Q-DASH score was 28.6±14.3 in the good group and 73.0±6.2 in the poor group; this was significantly higher in the poor group than in the good group (Mann-Whitney U test, P=0.22). These results revealed that the poor group had a significantly lower body weight and more patients were classified as AO type C than the good group. The Q-DASH score was significantly lower in the good group than in the poor group (Table I).

In the good group, the duration from injury to surgery was 9.1±4.4 days, the direction of fracture dislocation was volar in 32.6% of patients and dorsal in 67.4% of patients, the RI was 13.5±9.4˚, VT was -6.5±20.3˚ and UV was 1.3±2.4 mm. In the poor group, the duration from injury to surgery was 7.6±5.4 days, the fracture dislocation direction was volar in 12.5% of patients and dorsal in 87.5% of patients, the RI was 6.8±11.3˚, VT was -23.8±20.1˚ and UV was 4.2±3.5 mm. At the time of injury, the VT was significantly lower in the poor group (P=0.02) and UV was significantly higher (P=0.01). There were no other significant differences between the groups (Table II). At 1 month following VLP fixation, in the good group, the RI was 21.6±6.5˚, VT was 10.6±4.8˚ and UV was -0.3±1.5 mm; the wrist range of motion was 53.3±15.8˚ in flexion, 54.3±13.9˚ in extension, 78.1±11.0˚ in pronation and 75.3±13.9˚ in supination; the grip strength ratio was 43.8±19.9% and the VAS score was 2.4±1.7. At 1 month following VLP fixation, in the poor group, the RI was 22.5±3.8˚, VT was 11.9±3.5˚, UV was -1.0±1.0 mm, the wrist range of motion was 50.6±12.4˚ in flexion, 43.8±15.8˚ in extension, 74.4±11.5˚ in pronation and 69.4±17.6˚ in supination; the grip strength ratio was 17.2±5.4% and the VAS score was 5.6±1.5. The grip strength ratio was significantly lower in the poor group (P<0.001) and the patients had more pain (P<0.001) than the good group (Table III).

Four factors were extracted as factors related to the poor group: VT and UV at the time of injury, and grip strength ratio and VAS at 1 month following VLP fixation. From these four factors, two factors were each combined and six-way logistic regression analysis was performed (Table IV). As a result, all four factors were found to be independently associated with the poor Q-DASH score group.

## Discussion

The aim of the present study was to identify clinical findings involved in a poor Q-DASH score in the very early post-operative period of 1 month following VLP fixation. In the present study, VT and UV at the time of injury, and the VAS score and grip strength ratio at 1 month following VLP fixation were the clinical findings that contributed to a poor Q-DASH score at 1 month following VLP fixation. Interventions to improve these clinical findings may lead to an immediate return to activity following VLP fixation. In distal radius fractures, it has been reported that distal radius fractures with large VT and UV displacement at the time of injury are more likely to develop triangular fibrocartilage complex (TFCC) injury (14). It is also known that patients with soft tissue injury, such as TFCC injury tend to have poor post-operative outcomes following VLP fixation (15). In other words, previous reports have suggested that VT and UV at the time of injury are associated with post-operative outcomes following VLP fixation. It has also been reported that post-operative pain tends to hinder rehabilitation, resulting in reduced patient satisfaction (16). Furthermore, Beumer and Lindau (17) investigated the post-operative grip strength ratio, range of motion of the wrist joint and radiographic parameters in patients with hand and wrist trauma, and analyzed the factors influencing the acquisition of postoperative activities of daily life. As a result, it was reported that the post-operative grip strength ratio had the greatest influence on the level of activities of daily life (17). Based on these findings and the results of the present study, fracture displacement (VT and UV), post-operative pain and post-operative grip strength may be key factors for immediate return to activity.

As regards the alignment of the radius at the time of injury, the present study found a greater amount of UV and VT displacement in the poor group than the good group. It has been reported that the extent of comminution and the amount of fracture displacement are negatively associated with bone mineral density (18). Clayton *et al* (19) also reported that impact energy and wrist position at the time of injury were associated with the amount of fracture displacement. Among the factors that influence the severity of fracture type, bone mineral density is a factor that can potentially be treated by medical staff. However, following distal radius fracture injury, the diagnosis of osteoporosis is rare and the intervention rate for treatment is low (~10%) (20). Addressing osteoporosis treatment alongside the management of distal radius fractures poses a challenge for medical staff. Moreover, the potential of mesenchymal stem cell-derived extracellular vesicles and exosomes derived from young plasma as a novel treatment for osteoporosis focusing on osteogenic differentiation has also recently been reported (21,22). The challenge for medical professional is to be proactive in the treatment of osteoporosis, including new therapies, to prevent an increase amount of displacement in the patient with distal radius fractures.

The Q-DASH score was developed as a shortened version of the DASH Outcome measure. Instead of the 30 items of the DASH Outcome measure, the Q-DASH score uses 11 items to measure physical function and symptoms in people with any or multiple musculoskeletal disorders of the upper extremity. Of the 11 items, nine are questions that involve various factors, such as range of motion and grip strength and their impact on social activities, work and sleep, whereas the remaining two items ask about the severity of pain (9). Hence, interventions for pain may alter the evaluation of two of the 11 items. In the present study, the VAS score of the good group was 2.4±1.7, whereas that of the poor group was 5.6±1.5. These findings suggest that appropriate post-operative pain management enables an immediate return to activity. Comparing the patient background in the two groups, there were significantly more type C fractures in the poor group compared with the good group (Table I). A previous study suggested that intra-articular fractures result in more severe postoperative pain than extra-articular fractures (23). Therefore, the type of fracture may be a key factor in post-operative pain.

Previous studies have reported intraoperative and post-operative pain management methods. Egol *et al* (16) reported that during surgery, subclavian blocks provide more effective post-operative analgesia than general anesthesia alone. Luo *et al* (24) reported that opioids provide more effective pain relief during rehabilitation than COX inhibitors alone. In numerous centers, intraoperative and post-operative pain management methods are determined by the surgeon and anesthesiologist, and there is no unified protocol for pain management. Pain management is insufficient in the majority of cases (25). It is critical to understand that pain management is a key factor in improving the Q-DASH score and establishing appropriate pain management. Furthermore, it has recently been reported that platinum cluster-loaded-mesoporous polydopamine nanoparticle and QX-314-loaded fibrin gel (Pt@MPDA/QX314@Fibrin), a therapeutic agent for chronic diabetic ulcer wounds, has a pain-relieving effect by inactivating glial cells in the dorsal root ganglion. The development of such novel therapeutic agents with the potential to relieve post-operative pain is desirable (26). Post-operative pain control is a key factor for immediate return to activity in patients with distal radius fractures, and it is crucial to establish appropriate pain management, including new therapies.

In the present study post-operative grip strength was also found to be critical for immediate return to activity (Table IV). A previous study suggested that grip strength was associated with the walking ability following distal radius fracture (27). Furthermore, grip strength has been shown to be associated with the Q-DASH score in individuals with or without upper limb disorders (17,28). Based on these findings, the results of the present study suggested that grip strength was associated with the Q-DASH score, an indicator of return to activity, even in the early post-operative stage of 1 month following surgery. However, in rehabilitation following VLP fixation, although range of motion training is actively performed from an early stage, there are concerns about grip strength training due to excessive load on the fracture site (29). Grip strength training following VLP fixation surgery is currently challenging. On the other hand, Kaji *et al* (30) developed a rehabilitation program that significantly improves the post-operative Q-DASH score without causing loss of correction. Specifically, in their study, the patients began grip strength training under occupational therapist supervision using a 0.7 kg gripper on the 14th post-operative day, followed by training using a 1.4 kg load on the 21st day and a 2.3 kg load on the 28th day. Training was performed once a day for 20 min during hospitalization, and two to three times a week for 20 min each time following discharge from the hospital. The findings of the present study support this rehabilitation approach for immediate return to activity following VLP fixation. Aggressive grip strength rehabilitation in the early post-operative period following VLP fixation is critical for immediate return to activity.

The present study has several limitations. First, all 8 patients in the poor group had an AO classification of type C. Type C fractures are sometimes associated with poor outcomes due to the loss of correction and limited range of motion following VLP fixation (31,32). However, in the present study, there were no significant differences in radiographic parameters or range of motion at 1 month post-operatively between the good and poor groups, indicating that the previously reported factors for poor outcomes had been overcome. Second, although there were 270 cases during the study period, only 95 cases with available data at 1 month post-operatively were selected for inclusion. In the outpatient clinic for the patients with distal radius fracture post-operatively, the evaluation of clinical outcomes were performed normally at 3, 6 and 12 months post-operatively at the authors' hospital. Since the present study analyzed clinical outcomes at 1 month post-operatively, the subjects were 95 patients (~35% of all 270 patients) who had been evaluated at 1 month post-operatively. Thus, there may have been inclusion bias. Third, the number of cases was limited. Further studies with a larger number of cases and data are thus warranted.

In conclusion, in the present study, a large amount of fracture displacement at the time of injury was found to be a factor that interfered with immediate return to activity following VLP fixation. Fracture displacement at the time of injury was reported to be associated with bone mineral density. Thus, intervention to treat osteoporosis is critical. Furthermore, post-operative pain management and grip strength training were found to be key factors associated with immediate return to activity. Thus, aggressive intervention, such as grip strength training and pain management, may be necessary for the immediate return to activity following VLP fixation.

## Figures and Tables

**Table I:** Comparison of the background of the patients in the good and poor groups.

Characteristic	Good group (n=86)	Poor group (n=8)	P-value
Age, years	61.9±14.7	63.6±15.2	0.95
Sex			
Male	22	0	0.19
Female	64	8	
Height (cm)	158.2±14.7	153.0±8.0	0.33
Weight (kg)	58.6±10.1	50.5±8.2	0.03
BMI (kg/m^2^)	22.9±2.9	21.6±3.3	0.22
Injury energy^a^			
High	19	2	NS
Low	67	6	
AO classification			
Type A	18	0	<0.01
Type B	5	0	
Type C	63	8	
Q-DASH score	28.6±14.3	73.0±6.2	<0.01

The data for age, height, weight, BMI and Q-DASH score were analyzed using the Mann-Whitney U test. Data for sex, injury energy and AO classification were analyzed using Fisher's exact test.

^a^Injury energy was categorized as high and low energy (high energy injury pertains to injury sustained by a vehicle accident or falling from a height; low energy injury pertains to injury sustained falling down). BMI, body mass index; AO classification, Arbeitsgemeinschaft für Osteosynthesefragen classification; Q-DASH score, quick disabilities of the arm, shoulder and hand score, NS, not significant.

**Table II:** Comparison of the evaluation items at the time of injury between the good and poor groups.

Parameter	Good group	Poor group	P-value
Duration from injury to surgery (days)	9.1±4.4	7.6±5.4	0.23
Direction of fracture dislocation (%)			
Volar	32.6	12.5	0.43
Dorsal	67.4	87.5	
Radiographic parameters			
Radial inclination (˚)	13.5±9.4	6.8±11.3	0.06
Volar tilt (˚)	-6.5±20.3	-23.8±20.1	0.02
Ulnar variance (mm)	1.3±2.4	4.2±3.5	0.01

The data for the direction of fracture dislocation were analyzed using Fisher's exact test. The data for the other evaluation items were analyzed using the Mann-Whitney U test.

**Table III:** Comparison of evaluation items at 1 month post-operatively in the good and poor groups.

Parameter	Good group	Poor group	P-value
Radiographic parameters			
Radial inclination (˚)	21.6±6.5	22.5±3.8	0.61
Volar tilt (˚)	10.6±4.8	11.9±3.5	0.50
Ulnar variance (mm)	-0.3±1.5	-1.0±1.0	0.13
Wrist joint range of motion			
Flexion (˚)	53.3±15.8	50.6±12.4	0.62
Extension (˚)	54.3±13.9	43.8±15.8	0.06
Pronation (˚)	78.1±11.0	74.4±11.5	0.23
Supination (˚)	75.3±13.9	69.4±17.6	0.36
Grip strength ratio (%)	43.8±19.9	17.2±5.4	<0.001
VAS score	2.4±1.7	5.6±1.5	<0.001

The data for all the evaluation items were analyzed using the Mann-Whitney U test. VAS, visual analog scale.

**Table IV:** Results of logistic regression analysis.

A, Ulnar variance and volar tilt				
Parameter	Good group	Poor group	OR (95% CI)	P-value
Ulnar variance	1.3±2.4	4.2±3.5	1.41 (1.07-1.89)	0.01
Volar tilt	-6.5±20.3	-23.8±20.1	0.96 (0.92-1.00)	0.05
B, Ulnar variance and grip strength ratio				
Ulnar variance	1.3±2.4	4.2±3.5	1.41 (1.01-1.97)	0.02
Grip strength ratio	43.8±19.9	17.2±5.4	0.87 (0.77-0.97)	<0.01
C, Ulnar variance and VAS score				
Ulnar variance	1.3±2.4	4.2±3.5	1.40 (1.01-1.95)	0.04
VAS	2.4±1.7	5.6±1.5	2.18 (1.36-3.50)	<0.01
D, Volar tilt and VAS score				
Volar tilt	-6.5±20.3	-23.8±20.1	0.93 (0.88-0.99)	<0.01
VAS	2.4±1.7	5.6±1.5	3.07 (1.60-5.87)	<0.01
E, Grip strength ratio and VAS score				
Grip strength ratio	43.8±19.9	17.2±5.4	0.88 (0.77-1.00)	<0.01
VAS	2.4±1.7	5.6±1.5	2.14 (1.22-3.73)	<0.01
F, Volar tilt and grip strength ratio				
Volar tilt	-6.5±20.3	-23.8±20.1	0.95 (0.91-1.00)	0.02
Grip strength ratio	43.8±19.9	17.2±5.4	0.86 (0.78-0.96)	<0.01

OR, odds ratio; CI, confidence interval; VAS, visual analog scale.

## Data Availability

The datasets used and/or analyzed during the current study are available from the corresponding author on reasonable request.
